# Prevalence and Abundance of Beta-Lactam Resistance Genes in Hospital Wastewater and Enterobacterales Wastewater Isolates

**DOI:** 10.3390/tropicalmed8040193

**Published:** 2023-03-27

**Authors:** Dewi Santosaningsih, Aulia Putri Fadriyana, Nathanael Ibot David, Irene Ratridewi

**Affiliations:** 1 Department of Clinical Microbiology, Faculty of Medicine, Universitas Brawijaya, Malang 65145, Indonesia; 2 Department of Clinical Microbiology, Dr. Saiful Anwar Hospital, Malang 65112, Indonesia; 3 Biomedical Sciences Master Program, Faculty of Medicine, Universitas Brawijaya, Malang 65145, Indonesia; 4 Department of Pediatrics, Faculty of Medicine, Universitas Brawijaya, Malang 65145, Indonesia; 5 Department of Pediatrics, Dr. Saiful Anwar Hospital, Malang 65112, Indonesia

**Keywords:** antimicrobial resistance, environment, Indonesia

## Abstract

Antimicrobial resistance may develop in nature including in hospital wastewater through horizontal genetic transfer. Few studies were conducted on the antimicrobial resistance genes in hospital wastewater and wastewater isolates in Indonesia. The prevalence and abundance of beta-lactam resistance genes in hospital wastewater and Enterobacterales wastewater isolates were investigated. Twelve wastewater samples were collected from an influent wastewater treatment plant. *Escherichia coli* and *Klebsiella pneumoniae* were isolated from the wastewater samples by culture-based methods. DNA was extracted from wastewater samples and the isolates. Nineteen beta-lactam resistance genes were tested by a high throughput qRT-PCR method. *bla_GES_* and *bla_TEM_* were the most abundant genes detected in hospital wastewater and *Escherichia coli*, respectively (*p* < 0.001). The relative abundance of *bla_CMY_2_*, *bla_CTX-M5_*, *bla_CTX-M8_*, *bla_GES_*, *bla_NDM_*, and *bla_SHV11_* in *Klebsiella pneumoniae* was higher than in the wastewater and *Escherichia coli* (*p* < 0.001; *p* = 0.006; *p* = 0.012; *p* < 0.001; *p* = 0.005; *p* < 0.001). *Klebsiella pneumoniae* might be associated with resistance to piperacillin/tazobactam, ceftriaxone, and cefepime (*p* < 0.001; *p* = 0.001; *p* < 0.001). In conclusion, ESBL genes showed higher abundance than carbapenemase genes in hospital wastewater samples. The ESBL-producing bacteria that were predominantly found in hospital wastewater may originate from clinical specimens. The culture-independent antibiotic resistance monitoring system might be developed as an early warning system for the increasing beta-lactam resistance level in clinical settings.

## 1. Introduction

Antimicrobial resistance is an important public health problem worldwide issued by the World Health Organization [1]. The high prevalence of antimicrobial resistance leads to the high mortality and healthcare cost either in developed countries or low-middle income countries [2,3]. Previous studies reported that more than 2.8 million antimicrobial-resistant infections and 29,000 mortality cases occur each year in the United States [2,4].

Antibiotic resistance is high among Gram negative Enterobacterales nosocomial pathogens *Escherichia coli* and *Klebsiella pneumoniae* [5]. A national surveillance on antimicrobial resistance in Indonesia reported that the third generation cephalosporins resistant- *Escherichia coli* and *Klebisella pneumoniae* were the most WHO priority pathogens encountered in clinical specimens [6]. It might be associated with the high use of ceftriaxone as the empirical antibiotic therapy in Indonesian hospitals [7,8].

Beta-lactam, as broad-spectrum antibiotics, are the most commonly prescribed antibiotics in clinical settings. Thus, the extensive use of these antibiotics applies selective pressure towards human microbiota and pathogens, increasing the risk of developing resistant strains and limitation of antibiotic therapy [9].

There are several mechanisms of beta-lactam resistance such as inactivation by beta-lactamase production, decreased penetration to target sites, changes in penicillin-binding protein of target sites, and drug efflux through specific pumping mechanisms [10]. The beta-lactamase enzymes are encoded by several resistance genes both chromosomal DNA and plasmid DNA [11]. Spontaneous mutation and horizontal gene transfer play a role in inducing beta-lactamase production on chromosomal DNA and plasmid DNA, respectively [11].

Horizontal gene transfer greatly contributes to the rapid spread of antibiotic resistance [12]. The mechanism of horizontal gene transfer provides a wide range of opportunities for bacteria that exist in the same ecosystem to express a resistance toward a certain group of antibiotics. This rapid spread of antibiotic resistant genes allows much larger epidemic of antibiotic resistant bacteria to exist in future settings [13]. The activity of such gene transfer remains unrecognized in low- and middle-income countries allowing the development of antibiotic resistant bacteria in those ecosystems yet. Nevertheless, the importance of such detection could not be underpinned, making ways of detecting such gene transfer activity in certain high-risk environment with various easier detection methods is of topmost importance [14].

Hospital wastewater is regarded as a hotspot for antimicrobial resistance, allowing antibiotic resistance genes to be transferred horizontally between pathogens and commensal bacteria [5,15,16]. The abundance of ARGs in pathogens, particularly those causing healthcare associated infections, such as Enterobacterales isolated from hospital wastewater is unclear. *Escherichia coli* and *Klebsiella pneumoniae* are major nosocomial pathogens that deserve to be tested for its abundance of antibiotic resistance genes.

To present the aforementioned data regarding the current conditions on horizontal gene transfer on high-risk environment settings such as hospital wastewater would prove to provide a great benefit to monitor the possibility of potential outbreak. Especially in developing countries, the availability of this particular data would increase the awareness of in-depth monitoring on antibiotic resistance activity.

Majlander J. et al., 2021, reported a novel wastewater-based monitoring of antibiotic resistance including beta-lactam antibiotic resistance, which allows comparison of beta-lactam antibiotic resistance profile over time. In addition, the correlation between *bla_KPC_* and *Klebsiella pneumoniae* in wastewater samples was reported. Such methods could reflect both clinical activity and the relationship between antibiotic use and the relative abundance of genes encoding antibiotic resistance in hospital wastewater. Therefore, the culture-independent antibiotic resistance monitoring system would provide real-time data of the increasing beta-lactam resistance level [17].

Urgency of the availability of such monitoring method in low- and middle- income countries need to be presented in an assured and massive manner to provide sufficient data in real world setting towards future environmental policy [14]. The one health approach in hospital environments, either wards or wastewater, is expected to support antibiotic resistance control in hospital settings. To our knowledge, study on antimicrobial resistance in hospital wastewater in Indonesia is scarce [18,19]. The analysis of antibiotic resistance genes in nosocomial pathogens obtained from hospital wastewater has not yet been performed. This study aimed to measure the beta-lactam resistance genes through two different approaches, including directly from hospital wastewater and from two nosocomial pathogens (*Escherichia coli* and *Klebsiella pneumoniae*) isolated from the hospital wastewater, in a referral hospital in Indonesia. The correlation of beta-lactam resistance genes according to the samples collected each week, and the difference between the relative abundance of the beta-lactam resistance genes in hospital wastewater and nosocomial pathogens were evaluated in this study.

## 2. Materials and Methods

### 2.1. Setting and Sample Collection

The study was carried out in the Dr. Saiful Anwar Hospital in Malang, Indonesia, which is a 700-bed referral hospital. Wastewater samples were collected from an influent aerobic wastewater treatment plant containing untreated wastewater flowing from a mixture of wards, laundry, and kitchen. Sampling was performed twice a week randomly for six weeks from October 2021 until November 2021 (weeks 40–45); in total, we collected 12 hospital wastewater samples. Samples were collected using clean equipment by trained sampling personnel with personnel protective equipment. Grab samples of one liter of wastewater were collected in sterile bottles and kept cold for the 30 min it took to reach the lab.

### 2.2. Wastewater Filtration

The wastewater filtration method was carried out as previously described [17]. As soon as the wastewater sample arrived at the laboratory, 100 mL wastewater sample was concentrated directly into autoclaved poly-ethersulfone (PES) hydrophilic membranes 0.2 m using a Nalgene Rapid-Flow disposable filter unit (Thermo Fisher Scientific, MA, USA). After being transferred to PowerWater bead tubes, the filters were stored at −20 °C until the DNA was extracted.

### 2.3. Cultivation and Identification of Target Nosocomial Pathogens

We screened *Escherichia coli* and *Klebsiella pneumoniae* as target nosocomial pathogens from hospital wastewater samples. Fifty milliliters of hospital wastewater from sample bottle were transferred into conical centrifuge tube. Then, the samples were concentrated by centrifugation with 3000 rpm speed for 10 min to generate intact bacterial pellets.

A loop of bacterial pellets was inoculated to Eosin Methylene Blue (EMB) agar (Oxoid, Basingstoke, UK) and MacConkey agar (Oxoid, Basingstoke, UK), at 37 °C overnight to get cultural characteristics of *Escherichia coli* and *Klebsiella pneumoniae* colonies, respectively. Colonies showing metallic sheen in EMB agar and mucoid lactose fermenter colonies in MacConkey agar were suspected. In case the colonies were not isolated, the subculture was carried out for obtaining pure culture.

Vitek2 system was used to identify *Escherichia coli* and *Klebsiella pneumoniae* and performed antibiotic susceptibility testing (bioMérieux, Marcy l’Etoile, France). Beta-lactam antibiotics tested included ampicillin, ampicillin/sulbactam, piperacillin/tazobactam, cefazolin, ceftazidime, ceftriaxone, cefepime, and meropenem (Table S1). Antibiotic susceptibility test results were interpreted by the Clinical Laboratory Standard Institute 2022 guideline.

### 2.4. DNA Isolation

DNA isolation was carried out through two approaches. First, the DNA was directly isolated from the PES membrane in PowerWater bead tubes using the DNeasy PowerWater Kit (Qiagen, Venlo, The Netherlands) based on the manual instructions. It represented the DNA encountered in the hospital wastewater. Second, the DNA was extracted from the targeted nosocomial pathogens isolated from the hospital wastewater sample using DNeasy Blood and Tissue Kit (Qiagen, Venlo, The Netherlands). DNA was spectrophotometrically measured for both quantity and quality using NanoDrop One (Thermo Fisher Scientific, Waltham, MA, USA). Prior to delivery to Resistomap, Finland, DNA was kept at −20 °C.

### 2.5. High-Throughput Quantitative PCR Analysis

The DNA extract of 29 samples: 12 samples from hospital wastewater (ww1, ww2, ww3, ww4, ww5, ww6, ww7, ww8, ww9, ww10, ww11, ww12) and 17 samples from nosocomial pathogens obtained from the hospital wastewater (Ec1, Ec2, Ec5, Ec6, Ec7, Ec8, Ec9, Kp2, Kp3, Kp4, Kp5, Kp6, Kp7, Kp8, Kp9, Kp11, and Kp12) were transported to Resistomap Oy (Helsinki, Finland) for further genomic analysis as previously described [14]. First, 384 primers set 2.0 was used to screen the DNA samples for ARGs, followed by 198 primers set selection with a positive detection and one primer set for the 16s rRNA gene. From this set, 72 primers set were selected; however, 19 beta-lactam resistance genes including *bla_CARB_*, *bla_CMY_2_*, *bla_CMY2_*, *bla_CTX-M_5_*, *bla_CTX-M_8_*, *bla_GES_*, *bla_IMI_*, *bla_KPC_*, *bla_KPC_2_*, *bla_MOX_/bla_CMY_*, *bla_NDM_*, *bla_OXA48_*, *bla_OXA51_*, *bla_SHV11_*, *bla_SME_*, *bla_TEM_*, *bla_VEB_*, *bla_VIM_*, and *cfxA* were analyzed. Information regarding the primers is presented in Table S2. The beta-lactam resistance genes were chosen according to the common antibiotics used in Dr. Saiful Anwar Hospital. The 16s rRNA gene was used as the positive control and to normalize the abundance of detected genes in the samples.

The 72 primers sets were quantified using a SmartChip Quantitative PCR (HT-qPCR) system, a SmartChip Real-Time PCR system (Takara Bio, Mountain View, CA, USA). The SmartChip Real-Time PCR has 5184 reaction wells in a 100 nL volume that contain 1X SmartChip TB Green Gene Expression Master Mix (Takara Bio, Japan), nuclease-free PCR-grade water, 300 nM of each primer, and a 2 ng/L DNA template mixture. A 10 min denaturation at 95 °C was followed by 40 cycles of 30 s at 95 °C and 30 s at 60 °C. Each primer set was subjected to melting curve analysis. As the detection limit, a Ct value of 27 was chosen. Amplicons with non-specific melting curves and multiple peaks were ruled out [20].

### 2.6. Statistical Analysis and Visualization Using ResistApp

As previously described [17], data from the High-Throughput SmartChip quantitative PCR system was processed and analyzed using a digital platform, ResistApp (Resistomap, Finland). SPSS 26.0 was used to compare the relative abundance of beta-lactam antibiotic resistance genes from hospital wastewater to those obtained from nosocomial pathogens encountered in hospital wastewater. *p* < 0.05 was considered significant.

## 3. Results

### 3.1. Monitoring of Beta-Lactam Resistance Genes in Hospital Wastewater and Nosocomial Pathogens by Time

Monitoring 19 beta-lactam resistance genes in the hospital wastewater was carried out for six weeks in this study. Relative abundance of beta-lactam resistance genes detected over time in hospital wastewater samples, *Escherichia coli*, and *Klebsiella pneumoniae* were presented in Table 1.

Except for two genes, *bla_IMI_* and *bla_SME_*, all targeted beta-lactam resistance genes were found in hospital wastewater over time. We discovered 12 beta-lactam resistance genes that were always present with varying relative abundance in the hospital wastewater after six weeks of monitoring, out of the 17 beta-lactam resistance genes detected over time. Several wastewater samples lacked five carbapenem resistance genes: *bla_KPC_*, *bla_KPC2_*, *bla_OXA48_*, *bla_OXA51_*, and *bla_VIM_*. Within six weeks of monitoring, however, no beta-lactam resistance genes were always detected in *Escherichia coli* and *Klebsiella pneumoniae* isolated from hospital wastewater. *Escherichia coli* and *Klebsiella pneumoniae* revealed only eight and seven beta-lactam resistance genes, respectively (Table 1). The relative abundance of beta-lactam resistance genes either carbapenem resistance genes or extended spectrum beta-lactamase (ESBL) genes was not significantly different by the time of sampling (Kruskal–Wallis analysis; *p* = 0.142).

### 3.2. Comparison of the Relative Abundance of Beta-Lactam Resistance Genes in Hospital Wastewater, Escherichia coli, and Klebsiella pneumoniae

The relative abundance of each beta-lactam resistance genes in hospital wastewater, *Escherichia coli*, and *Klebsiella pneumoniae* within six weeks of monitoring was compared. One way ANOVA analysis showed that the relative abundance of *bla_GES_* was significantly higher compared to other beta-lactam resistance genes found in the wastewater samples (*p* < 0.001). Furthermore, *bla_TEM_* was significantly most abundant among beta-lactam resistance genes in *Escherichia coli* (*p* < 0.001) (Table 2).

Table 3 shows that among ESBL genes, *bla_GES_* reached the highest abundance only in hospital wastewater (1 × 10^−2^ copies/16s rRNA gene copies), whereas *bla_TEM_* was the most abundant in *Escherichia coli* (4 × 10^−2^ copies/16s rRNA genes copies) and *Klebsiella pneumoniae* (1 × 10^−4^ copies/16s rRNA genes copies). The median of relative abundance of each beta-lactam resistance genes in hospital wastewater, *Escherichia coli*, and *Klebsiella pneumoniae* during the study period was analyzed by Kruskal–Wallis. The relative abundance of all beta-lactam resistance genes was significantly different among hospital wastewater samples, *Escherichia coli*, and *Klebsiella pneumoniae* except for *bla_OXA48_* and *bla_TEM_* (Table 3).

We found that *bla_CMY_2_*, *bla_CTX-M5_*, *bla_CTX-M8_*, *bla_GES_*, *bla_NDM_*, and *bla_SHV11_* were more abundant in *Klebsiella pneumoniae* isolates compared to the wastewater samples and *Escherichia coli* (*p* < 0.001; *p* = 0.006; *p* = 0.012; *p* < 0.001; *p* = 0.005; *p* < 0.001). *bla_CARB_*, *bla_KPC_*, *bla_OXA48_*, *bla_OXA51_*, *bla_VIM_*, and *cfxA* were detected in wastewater samples but they were not found in *Escherichia coli* and *Klebsiella pneumoniae* isolated from hospital wastewater (Table 3).

### 3.3. Antibiotic Susceptibility Profile of Escherichia coli and Klebsiella pneumoniae Isolated from Hospital Wastewater

We found seven *Escherichia coli* and 10 *Klebsiella pneumoniae* isolates in 12 hospital wastewater samples. The antibiotic susceptibility test was carried out prior to further ARGs analysis. The antibiotic susceptibility test showed multidrug resistant *Klebsiella pneumoniae* isolated from hospital wastewater, which were resistant to all antibiotics tested except meropenem. All isolates were susceptible to meropenem. Statistical analysis showed that *Klebsiella pneumoniae* might be associated with the resistance to piperacillin/tazobactam (*p* < 0.001), ceftriaxone (*p* = 0.001), and cefepime (*p* < 0.001) (Table 4).

## 4. Discussion

To our knowledge, this is the first study that analyzed the presence and abundance of antimicrobial resistance genes (ARGs) in hospital wastewater samples compared to nosocomial pathogens isolated from hospital wastewater samples in Indonesia. In addition, this study specifically analyzed the various beta-lactam resistance genes in hospital wastewater samples and nosocomial pathogens isolated from the hospital wastewater.

Through our analysis, extended spectrum beta-lactamase (ESBL) genes and carbapenem resistance genes were present in hospital wastewater samples over time. *bla_GES_* was the most abundant beta-lactam resistance genes found in hospital wastewater (*p* < 0.001). In concordance with the hospital wastewater samples, we detected two *Klebsiella pneumoniae* isolates bearing high abundance of *bla_GES_*. *bla_GES_* is a transferable gene located in plasmid encoding carbapenem resistant to *Pseudomonas aeruginosa* and *Klebsiella pneumoniae* [21]. Although the *bla_GES_* was detected over time in wastewater samples, the abundance of *bla_GES_* was lower than in *Klebsiella pneumoniae isolates*. Wastewater treatment processes might affect the presence of antibiotic resistance genes in wastewater samples [22]. Further study is required to investigate the correlation between *bla_GES_* in hospital wastewater and *Klebsiella pneumoniae* isolated from the hospital wastewater. Furthermore, the clinical activity may be represented by ARGs profile in hospital wastewater.

Antibiotic susceptibility profile showed higher resistant to piperacillin/tazobactam, ceftriaxone, and cefepime among *Klebsiella pneumoniae* than *Escherichia coli* isolated from hospital wastewater (*p* < 0.001, *p* = 0.001, and *p* < 0.001). The high resistance of *Klebsiella pneumoniae* in the hospital wastewater might be associated with the high prevalence of third generation cephalosporins clinical *Klebsiella pneumoniae* isolates as reported by a national surveillance on antimicrobial resistance in Indonesia [5]. The nosocomial pathogens encountered in wastewater samples may originate from clinical isolates. Therefore, hospital wastewater has higher risk of ARGs dissemination through horizontal gene transfer such as transduction, transformation, and conjugation [22,23].

The present study showed predominant ESBL genes including *bla_TEM_*, *bla_CTX-M5_*, and *bla_CTX-M8_*, which were encountered in *Escherichia coli* and *Klebsiella pneumoniae* isolated from wastewater samples. *bla_TEM_* was the most abundant among the ESBL genes in this study. This result is in concordance with the high prevalence of ESBL-producing *Escherichia coli* and *Klebsiella pneumoniae* among clinical cultures reported in the previous studies [24,25,26]. It is suggested that *Escherichia coli* and *Klebsiella pneumoniae* in the wastewater samples originated from the clinical specimens. Similar to *bla_GES_*, *bla_TEM_* is a transferable gene located in a plasmid encoding ESBL enzymes; therefore, they spread easily among different bacteria.

Carbapenemase genes, including *bla_CARB_*, *bla_KPC_*, *bla_OXA48_*, *bla_OXA51_*, and *bla_VIM_* were detected in hospital wastewater but not in *Escherichia coli* and *Klebsiella pneumoniae* isolated from the wastewater samples. It is aligned with the antibiotic susceptibility profile, presenting no meropenem resistant among *Escherichia coli* and *Klebsiella pneumoniae*. In accordance with the previous study, there was no association between the relative abundance of antibiotic resistance genes in wastewater samples to those in the clinical isolates [27,28].

In this study, *bla_IMI_* and *bla_SME_* genes were not detected either in hospital wastewater samples or in *Escherichia coli* and *Klebsiella pneumoniae* isolates over time. *bla_IMI_* and *bla_SME_* are carbapenemase genes encoding IMI and SME enzymes located in the chromosome of *Enterobacter cloacae* and *Serratia marcescens*, respectively [29]. Therefore, the *bla_IMI_* and *bla_SME_* genes are restricted in *Enterobacter* and *Serratia* genus due to the less transferable genes [21,30].

## 5. Conclusions

We reported the dynamics of the abundance of beta-lactam resistance genes in hospital wastewater and nosocomial pathogens in six weeks of monitoring. Higher abundances of *bla_GES_* in hospital wastewater and *bla_TEM_* in *Escherichia coli* and *Klebsiella pneumoniae* isolated from the hospital wastewater were detected than other beta-lactam resistance genes. Further investigation is required to evaluate the correlation between the ESBL genes in hospital wastewater and the prevalence of ESBL-producing *Escherichia coli* and *Klebsiella pneumoniae* obtained from clinical cultures. Therefore, a potential outbreak of ESBL-producing *Escherichia coli* and *Klebsiella pneumoniae* could be detected by hospital wastewater-based monitoring systems using culture independent methods.

This study had certain limitations. First, we used grab samples instead of composite samples leading to less representative ARGs data. Second, we screened two nosocomial pathogens including *Escherichia coli* and *Klebsiella pneumoniae* using conventional culture methods; therefore, the presence of other pathogens was not detected. Third, wastewater samples were collected from an influent wastewater treatment plant containing untreated wastewater flowing from a mixture of wards, laundry, and kitchen that might be influenced by detergent or disinfectant antimicrobial activities. Fourth, the present study was a pilot of monitoring ARGs in hospital wastewater in Indonesia; therefore, we started with a small-scale study. Further investigation with more hospitals involved is recommended.

## Figures and Tables

**Table 1:** Gene abundance of beta lactamase resistance genes relative to 16S rRNA in hospital wastewater samples and pathogens (copies/16s rRNA gene copies).

Gene	Wastewater											
	Week 40		Week 41		Week 42		Week 43		Week 44		Week 45	
	ww1	ww2	ww3	ww4	ww5	ww6	ww7	ww8	ww9	ww10	ww11	ww12
*blaCARB*	3 × 10^−3^	1 × 10^−3^	1 × 10^−2^	6 × 10^−3^	1 × 10^−3^	2 × 10^−3^	4 × 10^−3^	3 × 10^−3^	3 × 10^−3^	9 × 10^−3^	1 × 10^−2^	7 × 10^−3^
*blaCMY_2*	3 × 10^−3^	2 × 10^−3^	2 × 10^−3^	3 × 10^−3^	2 × 10^−3^	4 × 10^−3^	4 × 10^−3^	4 × 10^−3^	4 × 10^−3^	4 × 10^−3^	4 × 10^−3^	4 × 10^−3^
*blaCMY2*	7 × 10^−4^	3 × 10^−4^	2 × 10^−4^	4 × 10^−4^	1 × 10^−4^	6 × 10^−4^	1 × 10^−3^	1 × 10^−3^	6 × 10^−4^	3 × 10^−4^	3 × 10^−4^	5 × 10^−4^
*blaCTX-M_5*	2 × 10^−4^	1 × 10^−4^	2 × 10^−4^	6 × 10^−4^	6 × 10^−4^	2 × 10^−3^	9 × 10^−4^	1 × 10^−3^	2 × 10^−4^	2 × 10^−4^	-	1 × 10^−3^
*blaCTX-M_8*	3 × 10^−4^	3 × 10^−4^	5 × 10^−4^	1 × 10^−3^	1 × 10^−3^	4 × 10^−3^	1 × 10^−3^	2 × 10^−3^	6 × 10^−4^	5 × 10^−4^	3 × 10^−4^	3 × 10^−3^
*blaGES*	1 × 10^−2^	1 × 10^−2^	1 × 10^−2^	1 × 10^−2^	1 × 10^−2^	2 × 10^−2^	2 × 10^−2^	2 × 10^−2^	1 × 10^−2^	2 × 10^−2^	4 × 10^−2^	3 × 10^−2^
*blaIMI*	-	-	-	-	-	-	-	-	-	-	-	-
*blaKPC*	-	-	-	2 × 10^−4^	-	2 × 10^−5^	5 × 10^−4^	-	2 × 10^−5^	-	-	9 × 10^−5^
*blaKPC_2*	6 × 10^−5^	-	-	8 × 10^−5^	-	-	2 × 10^−4^	1 × 10^−5^	2 × 10^−5^	-	-	2 × 10^−5^
*blaMOX/blaCMY*	7 × 10^−4^	5 × 10^−4^	2 × 10^−4^	7 × 10^−4^	2 × 10^−4^	6 × 10^−4^	1 × 10^−3^	7 × 10^−4^	5 × 10^−4^	4 × 10^−4^	3 × 10^−4^	9 × 10^−4^
*blaNDM*	8 × 10^−4^	2 × 10^−4^	6 × 10^−4^	4 × 10^−3^	5 × 10^−4^	9 × 10^−4^	2 × 10^−3^	1 × 10^−3^	8 × 10^−4^	9 × 10^−4^	9 × 10^−4^	2 × 10^−3^
*blaOXA48*	3 × 10^−5^	-	-	-	-	-	-	-	-	-	-	-
*blaOXA51*	-	2 × 10^−4^	4 × 10^−5^	1 × 10^−4^	3 × 10^−4^	1 × 10^−4^	-	2 × 10^−4^	2 × 10^−5^	-	-	4 × 10^−5^
*blaSHV11*	3 × 10^−4^	6 × 10^−4^	8 × 10^−4^	7 × 10^−4^	1 × 10^−3^	2 × 10^−3^	9 × 10^−4^	1 × 10^−3^	9 × 10^−4^	3 × 10^−4^	4 × 10^−5^	1 × 10^−3^
*blaSME*	-	-	-	-	-	-	-	-	-	-	-	-
*blaTEM*	3 × 10^−3^	2 × 10^−3^	3 × 10^−3^	6 × 10^−3^	7 × 10^−3^	1 × 10^−2^	8 × 10^−3^	7 × 10^−3^	3 × 10^−3^	3 × 10^−3^	1 × 10^−3^	1 × 10^−2^
*blaVEB*	4 × 10^−3^	1 × 10^−2^	4 × 10^−3^	4 × 10^−3^	8 × 10^−3^	8 × 10^−3^	1 × 10^−2^	2 × 10^−2^	5 × 10^−3^	5 × 10^−3^	5 × 10^−3^	1 × 10^−2^
*blaVIM*	2 × 10^−4^	-	2 × 10^−4^	2 × 10^−4^	2 × 10^−4^	6 × 10^−5^	4 × 10^−5^	9 × 10^−5^	1 × 10^−4^	5 × 10^−4^	4 × 10^−4^	4 × 10^−4^
*cfxA*	2 × 10^−3^	9 × 10^−4^	7 × 10^−4^	2 × 10^−3^	1 × 10^−3^	4 × 10^−3^	2 × 10^−3^	1 × 10^−3^	2 × 10^−3^	2 × 10^−3^	2 × 10^−3^	6 × 10^−3^
**Gene**	***Escherichia coli***											
	**Week 40**		**Week 41**		**Week 42**		**Week 43**		**Week 44**		**Week 45**	
	**Ec1**	**Ec2**			**Ec5**	**Ec6**	**Ec7**	**Ec8**	**Ec9**			
*blaCARB*	-	-	-	-	-	-	-	-	-	-	-	-
*blaCMY_2*	-	4 × 10^−4^	-	-	-	3 × 10^−4^	-	-	3 × 10^−4^	-	-	-
*blaCMY2*	-	-	-	-	-	-	1 × 10^−1^	-	-	-	-	-
*blaCTX-M_5*	-	4 × 10^−2^	-	-	-	-	-	5 × 10^−2^	-	-	-	-
*blaCTX-M_8*	-	9 × 10^−2^	-	-	7 × 10^−6^	-	-	1 × 10^−1^	-	-	-	-
*blaGES*	-	-	-	-	-	-	-	-	-	-	-	-
*blaIMI*	-	-	-	-	-	-	-	-	-	-	-	-
*blaKPC*	-	-	-	-	-	-	-	-	-	-	-	-
*blaKPC_2*	2 × 10^−5^	-	-	-	-	-	-	-	-	-	-	-
*blaMOX/blaCMY*	-	-	-	-	-	-	5 × 10^−2^	-	-	-	-	-
*blaNDM*	-	-	-	-	-	-	-	-	-	-	-	-
*blaOXA48*	-	-	-	-	-	-	-	-	-	-	-	-
*blaOXA51*	-	-	-	-	-	-	-	-	-	-	-	-
*blaSHV11*	-	-	-	-	-	-	-	-	-	-	-	-
*blaSME*	-	-	-	-	-	-	-	-	-	-	-	-
*blaTEM*	2 × 10^−1^	1 × 10^−1^	-	-	2 × 10^−1^	1 × 10^−1^	-	4 × 10^−1^	2 × 10^−1^	-	-	-
*blaVEB*	-	-	-	-	-	-	8 × 10^−2^	-	-	-	-	-
*blaVIM*	-	-	-	-	-	-	-	-	-	-	-	-
*cfxA*	-	-	-	-	-	-	-	-	-	-	-	-
**Gene**	***Klebsiella pneumoniae***											
	**Week 40**		**Week 41**		**Week 42**		**Week 43**		**Week 44**		**Week 45**	
		**Kp2**	**Kp3**	**Kp4**	**Kp5**	**Kp6**	**Kp7**	**Kp8**	**Kp9**		**Kp11**	**Kp12**
*blaCARB*	-	-	-	-	-	-	-	-	-	-	-	-
*blaCMY_2*	-	-	5 × 10^−6^	3 × 10^−4^	-	-	-	-	8 × 10^−2^	-	8 × 10^−6^	-
*blaCMY2*	-	-	-	-	-	-	-	-	-	-	-	-
*blaCTX-M_5*	-	-	-	3 × 10^−2^	-	-	-	-	1 × 10^−1^	-	-	-
*blaCTX-M_8*	-	-	-	7 × 10^−2^	-	-	9 × 10^−5^	-	3 × 10^−1^	-	-	2 × 10^−4^
*blaGES*	-	-	-	-	9 × 10^−1^	1	-	-	-	-	-	-
*blaIMI*	-	-	-	-	-	-	-	-	-	-	-	-
*blaKPC*	-	-	-	-	-	-	-	-	-	-	-	-
*blaKPC_2*	-	-	-	-	-	-	-	-	-	-	-	-
*blaMOX/blaCMY*	-	-	-	-	-	-	-	-	-	-	-	-
*blaNDM*	-	-	-	-	-	-	-	-	1 × 10^−1^	-	-	1 × 10^−4^
*blaOXA48*	-	-	-	-	-	-	-	-	-	-	-	-
*blaOXA51*	-	-	-	-	-	-	-	-	-	-	-	-
*blaSHV11*	-	2 × 10^−1^	-	-	-	-	-	-	-	-	-	-
*blaSME*	-	-	-	-	-	-	-	-	-	-	-	-
*blaTEM*	-	-	2 × 10^−1^	1 × 10^−1^	-	3 × 10^−4^	1 × 10^−1^	-	3 × 10^−1^	-	3 × 10^−1^	-
*blaVEB*	-	-	-	-	-	-	-	-	-	-	-	-
*blaVIM*	-	-	-	-	-	-	-	-	-	-	-	-
*cfxA*	-	-	-	-	-	-	-	-	-	-	-	-

**Table 2:** Beta-lactam resistance genes detected in hospital wastewater and pathogens isolated from hospital wastewater.

Samples	Number of Detected Genes	Beta-Lactam Resistance Genes																		
		*bla* *_CARB_*	*bla* *_CMY_2_*	*bla* *_CMY2_*	*bla* *_CTX-M_5_*	*bla* *_CTX-M_8_*	*bla* *_GES_*	*bla* *_IMI_*	*bla* *_KPC_*	*bla* *_KPC_2_*	*bla* *_MOX_*	*bla* *_NDM_*	*bla* *_OXA48_*	*bla* *_OXA51_*	*bla* *_SHV11_*	*bla* *_SME_*	*bla* *_TEM_*	*bla* *_VEB_*	*bla* *_VIM_*	cfxA
**Wastewater**																				
ww1	15																			
ww2	13																			
ww3	14																			
ww4	16																			
ww5	14																			
ww6	15																			
ww7	15																			
ww8	15																			
ww9	16																			
ww10	13																			
ww11	12																			
ww12	16																			
Mean ^a^		5 × 10^−3^	3 × 10^−3^	5 × 10^−4^	6 × 10^−4^	1 × 10^−3^	18 × 10^−3^	ND	6 × 10^−5^	3 × 10^−5^	5 × 10^−4^	1 × 10^−3^	2 × 10^−6^	9 × 10^−5^	8 × 10^−4^	ND	5 × 10^−3^	7 × 10^−3^	2 × 10^−4^	2 × 10^−3^
±SD		3 × 10^−3^	0.8 × 10^−3^	3 × 10^−4^	5 × 10^−4^	1 × 10^−3^	8 × 10^−3^	ND	14 × 10^−5^	6 × 10^−5^	3 × 10^−4^	1 × 10^−3^	2 × 10^−6^	9 × 10^−5^	4 × 10^−4^	ND	3 × 10^−3^	3 × 10^−3^	1 × 10^−4^	1 × 10^−3^
Pathogens																				
Ec1	2																			
Ec2	4																			
Ec5	2																			
Ec6	2																			
Ec7	3																			
Ec8	3																			
Ec9	2																			
Mean ^b^		ND	7 × 10^−5^	9 × 10^−3^	7 × 10^−3^	2 × 10^−2^	ND	ND	ND	2 × 10^−6^	4 × 10^−3^	ND	ND	ND	ND	ND	9 × 10^−2^	6 × 10^−3^	ND	ND
±SD		ND	1 × 10^−5^	30 × 10^−3^	16 × 10^−3^	4 × 10^−2^	ND	ND	ND	7 × 10^−6^	15 × 10^−3^	ND	ND	ND	ND	ND	13 × 10^−2^	22 × 10^−3^	ND	ND
Kp2	1																			
Kp3	2																			
Kp4	4																			
Kp5	1																			
Kp6	2																			
Kp7	2																			
Kp8	0																			
Kp9	5																			
Kp11	2																			
Kp12	2																			
Mean ^c^		ND	7 × 10^−3^	ND	1 × 10^−2^	3 × 10^−2^	16 × 10^−2^	ND	ND	ND	ND	1 × 10^−2^	ND	ND	2 × 10^−2^	ND	8 × 10^−2^	ND	ND	ND
±SD		ND	23 × 10^−3^	ND	4 × 10^−2^	9 × 10^−2^	39 × 10^−2^	ND	ND	ND	ND	4 × 10^−2^	ND	ND	6 × 10^−2^	ND	12 × 10^−2^	ND	ND	ND
Interpretation:																				
	≥1																			
	≥0.1																			
	≥0.01																			
	≥0.001																			
	≥0.0001																			
	≥0.00001																			
	white color indicates genes not detected																			

The colors indicate the gene abundances relative to 16s rRNA gene; ww = wastewater; Ec = *Escherichia coli*; Kp = *Klebsiella pneumoniae*; ND = not determined; ^a^ *p* < 0.001; ^b^ *p* < 0.001; ^c^ *p* = 0.153.

**Table 3:** Abundance of beta-lactam resistance genes in hospital wastewater and nosocomial pathogens isolated from hospital wastewater (copies/16s rRNA gene copies).

Genes	Gene Groups	Hospital Wastewater				*Escherichia coli*				*Klebsiella pneumoniae*				* *p* Value
		95% CI				95% CI				95% CI				
		N	LCI	Median	UCI	N	LCI	Median	UCI	N	LCI	Median	UCI	
*bla_CARB_*	Carbapenemase genes	12	3 × 10^−3^	3 × 10^−3^	7 × 10^−3^	0	-	-	-	0	-	-	-	<0.001
*bla_CMY_2_*	ESBL genes	12	3 × 10^−3^	3 × 10^−3^	4 × 10^−3^	3	−1 × 10^−5^	0	2 × 10^−4^	4	−8 × 10^−3^	0	2 × 10^−2^	<0.001
*bla_CMY2_*	ESBL genes	12	3 × 10^−4^	4 × 10^−4^	7 × 10^−4^	1	−1 × 10^−5^	0	2 × 10^−4^	0	-	-	-	<0.001
*bla_CTX-M_5_*	ESBL genes	11	2 × 10^−4^	0	1 × 10^−3^	2	−4 × 10^−3^	0	2 × 10^−2^	2	−1 × 10^−2^	0	4 × 10^−2^	0.006
*bla_CTX-M_8_*	ESBL genes	12	6 × 10^−4^	9 × 10^−4^	2 × 10^−3^	3	−5 × 10^−2^	0	2 × 10^−1^	4	−2 × 10^−2^	0	9 × 10^−2^	0.012
*bla_GES_*	ESBL genes	12	1 × 10^−2^	1 × 10^−2^	2 × 10^−2^	0	-	-	-	2	−8 × 10^−2^	0	4 × 10^−1^	<0.001
*bla_IMI_*	Carbapenamase genes	0	-	-	-	0	-	-	-	0	-	-	-	-
*bla_KPC_*	Carbapenamase genes	5	−3 × 10^−5^	0	2 × 10^−4^	0	-	-	-	0	-	-	-	0.004
*bla_KPC_2_*	Carbapenamase genes	6	−7 × 10^−6^	7 × 10^−6^	7 × 10^−5^	1	−2 × 10^−6^	0	6 × 10^−6^	0	-	-	-	0.005
*bla_MOX_/bla_CMY_*	ESBL genes	12	4 × 10^−4^	5 × 10^−4^	7 × 10^−4^	1	−5 × 10^−3^	0	1 × 10^−2^	0	-	-	-	<0.001
*bla_NDM_*	Carbapenamase genes	12	6 × 10^−4^	8 × 10^−4^	2 × 10^−3^	0	-	-	-	2	−1 × 10^−2^	0	3 × 10^−2^	0.005
*bla_OXA48_*	Carbapenamase genes	1	−3 × 10^−6^	0	9 × 10^−6^	0	-	-	-	0	-	-	-	0.368
*bla_OXA51_*	Carbapenamase genes	7	2 × 10^−5^	4 × 10^−5^	1 × 10^−4^	0	-	-	-	0	-	-	-	<0.001
*bla_SHV11_*	ESBL genes	12	5 × 10^−4^	8 × 10^−4^	1 × 10^−3^	0	-	-	-	1	−2 × 10^−2^	0	5 × 10^−2^	<0.001
*bla_SME_*	Carbapenamase genes	0	-	-	-	0	-	-	-	0	-	-	-	-
*bla_TEM_*	ESBL genes	12	3 × 10^−3^	4 × 10^−3^	8 × 10^−3^	6	2 × 10^−2^	4 × 10^−2^	2 × 10^−1^	6	1 × 10^−2^	1 × 10^−4^	2 × 10^−1^	0.885
*bla_VEB_*	Carbapenamase genes	12	5 × 10^−3^	6 × 10^−3^	1 × 10^−2^	1	−8 × 10^−3^	0	2 × 10^−2^	0	-	-	-	<0.001
*bla_VIM_*	Carbapenamase genes	11	1 × 10^−4^	1 × 10^−4^	3 × 10^−4^	0	-	-	-	0	-	-	-	<0.001
*cfxA*	Beta-lactamase genes	12	1 × 10^−3^	2 × 10^−3^	3 × 10^−3^	0	-	-	-	0	-	-	-	<0.001

N = number of samples; LCI = lower confidence interval; UCI = upper confidence interval; * median of relative abundance was analyzed by Kruskal–Wallis.

**Table 4:** Beta-lactam antibiotic susceptibility pattern of *Escherichia coli* and *Klebsiella pneumoniae* isolated from hospital wastewater.

No.	Isolates	Antibiotics							
		AMP	SAM	TZP	CZO	CAZ	CRO	FEP	MEM
1	Ec1	R	R	S	R	S	S	S	S
2	Ec2	R	R	S	R	S	S	S	S
3	Ec5	R	R	S	R	S	S	S	S
4	Ec6	R	S	S	R	S	S	S	S
5	Ec7	R	S	S	R	S	S	S	S
6	Ec8	R	R	S	R	S	S	S	S
7	Ec9	R	R	S	R	S	R	S	S
8	Kp2	R	R	R	R	R	R	R	S
9	Kp3	R	R	R	R	R	R	R	S
10	Kp4	R	R	R	R	R	R	S	S
11	Kp5	R	R	R	R	R	R	R	S
12	Kp6	R	R	R	R	R	R	R	S
13	Kp7	R	R	R	R	R	R	R	S
14	Kp8	R	R	R	R	R	R	R	S
15	Kp9	R	R	R	R	R	R	R	S
16	Kp11	R	R	R	R	R	R	R	S
17	Kp12	R	R	R	R	R	R	R	S
* p value		ND	0.154	<0.001	ND	<0.001	0.001	<0.001	ND

Ec = *Escherichia coli*; Kp = *Klebsiella pneumoniae*; AMP = Ampicillin; SAM = Ampicillin/Sulbactam; TZP = Piperacillin/Tazobactam; CZO = Cefazolin; CAZ = Ceftazidime; CRO = Ceftriaxone; FEP = Cefepime; MEM = Meropenem; S = susceptible; R = resistant; * Fisher exact test; ND = not determined.

## Data Availability

The data from this study are available upon request from the corresponding author.

## Supplementary Materials

The following supporting information can be downloaded at: https://www.mdpi.com/article/10.3390/tropicalmed8040193/s1, Table S1. Antibiotic Concentration using Vitek2 Gram-negative Susceptibility Panels (AST-GN95 and AST-XN09); Table S2. The 20 Primers targeting 19 beta-lactam resistance genes and the 16S rRNA gene. References [31,32] are cited in the Supplementary Materials.
