# Supplementary material for: Prevalence and Abundance of Beta-Lactam Resistance Genes in Hospital Wastewater and Enterobacterales Wastewater Isolates
Source: Trop Med Infect Dis. 2023 Mar 27;8(4):193. doi: 10.3390/tropicalmed8040193 (PMC10146145; doi:10.3390/tropicalmed8040193)
Supplement: Supplementary file 1 [file tropicalmed-08-00193-s001.zip › tropicalmed-2194979-supplementary.pdf]

Table S1. Antibiotic Concentration using Vitek2 Gram-negative Susceptibility Panels (AST-GN95 and AST-XN09)

Antimicrobial agent	MIC Calling range (μg/mL)
Ampicilin	2-32
Ampicilin/Sulbactam	2/1 - 32/16
Piperacilin/Tazobactam	4/4 - 128/4
Cefazolin	4-64
Ceftazidime	1 - 64
Ceftriaxone	0.25 - 64
Cefepime	0.12 - 32
Meropenem	0.25 - 16

Table S2. The 20 Primers targeting 19 beta-lactam resistance genes and the 16S rRNA gene.

**Assay**	***Gene***	**Forward Primer**	**Reverse Primer**
AY1	16S rRNA	GGGTTGCGCTCGTTGC	ATGGYTGTCGTCAGCTCGTG
AY446	*blaCARB*	TGATTTGAGGGATACGACAACTCC	CTGTAATACTCCGAGCACCAA
AY339	*blaCMY_2*	AAAGCCTCAT GGGTGCATAAA	ATAGCTTTTGTTTGCCAGCATCA
AY116	*blaCMY2*	GCGAGCAGCCTGAAGCA	CGGATGGGCTTGTCCTCTT
AY134	*blaCTX-M_5*	GCGATAACGTGGCGATGAAT	GTCGAGACGGAACGTTTCGT
AY147	*blaCTX-M_8*	CGTCACGCTGTTGTTAGGAA	CGCTCATCAGCACGATAAAG
AY125	*blaGES*	GCAATGTGCTCAACGTTCAAG	GTGCCTGAGTCAATTCTTTCAAAG
AY449	*blaIMI*	ACATCTACACCTGCAGCAGTAG	AATCGCTTGGTACGCTAGCA
AY440	*blaKPC*	GCCGCCAATTTGTTGCTGAA	GCCGGTCGTGTTTCCCTTT
AY153	*blaKPC_2*	GCCGCCGTGCAATACAGT	GCCGCCCAACTCCTTCA
AY101	*blaMOX/ blaCMY*	CTATGTCAATGTGCCGAAGCA	GGCTTGTCCTCTTTCGAATAGC
AY152	*blaNDM*	GGCCACACCAGTGACAATATCA	CAGGCAGCCACCAAAAGC
AY601	*blaOXA48*[a]	TGTTTTTGGTGGCATCGAT	GTAAMRATGCTTGGTTCGC
AY435	*blaOXA51*	CGACCGAGTATGTACCTGCTTC	TCAAGTCCAATACGACGAGCTA
AY438	*blaSHV11*	TTGACCGCTGGGAAACGG	TCCGGTCTTATCGGCGATAAAC
AY431	*blaSME*	GAGGAAGACTTTGATGGGAGGATTG	CGCTATATTGCAATGCAGCAGAAG
AY439	*blaTEM*	CGCCGCATACACTATTCTCAG	GCTTCATTCAGCTCCGGTTC
AY105	*blaVEB*	CCCGATGCAAAGCGTTATG	GAAAGATTCCCTTTATCTATCTCAG ACAA
AY129	*blaVIM*	GCACTTCTCGCGGAGATTG	CGACGGTGATGCGTACGTT
AY114	*cfxA*	TCATTCCTCGTTCAAGTTTTCAGA	TGCAGCACCAAGAGGAGATGT

Source primer: Stedtfeld RD, Guo X, Stedtfeld TM, Sheng H, Williams MR, Hauschild K, et al. Primer set 2.0 for highly parallel qPCR array targeting antibiotic resistance genes and mobile genetic elements. FEMS Microbiol Ecol 2018;94. https://doi.org/10.1093/femsec/fiy130. [a]Monteiro J, Widen RH, Pignatari ACC, Kubasek C, Silbert S. Rapid detection of carbapenemase genes by multiplex real-time PCR. J Antimicrob Chemother 2012; 67:906–9. https://doi.org/10.1093/jac/dkr563
